# Revealing the Dynamics of Thylakoid Membranes in Living Cyanobacterial Cells

**DOI:** 10.1038/srep19627

**Published:** 2016-01-21

**Authors:** Laura-Roxana Stingaciu, Hugh O’Neill, Michelle Liberton, Volker S. Urban, Himadri B. Pakrasi, Michael Ohl

**Affiliations:** 1 JCNS1, outstation at SNS, Oak Ridge National Laboratory, Oak Ridge, TN 37831, USA; 2 NScD, Oak Ridge National Laboratory, Oak Ridge, TN 37831, USA; 3 Department of Biology, Washington University, St. Louis, MO 63130, USA

## Abstract

Cyanobacteria are photosynthetic prokaryotes that make major contributions to the production of the oxygen in the Earth atmosphere. The photosynthetic machinery in cyanobacterial cells is housed in flattened membrane structures called thylakoids. The structural organization of cyanobacterial cells and the arrangement of the thylakoid membranes in response to environmental conditions have been widely investigated. However, there is limited knowledge about the internal dynamics of these membranes in terms of their flexibility and motion during the photosynthetic process. We present a direct observation of thylakoid membrane undulatory motion *in vivo* and show a connection between membrane mobility and photosynthetic activity. High-resolution inelastic neutron scattering experiments on the cyanobacterium *Synechocystis* sp. PCC 6803 assessed the flexibility of cyanobacterial thylakoid membrane sheets and the dependence of the membranes on illumination conditions. We observed softer thylakoid membranes in the dark that have three-to four fold excess mobility compared to membranes under high light conditions. Our analysis indicates that electron transfer between photosynthetic reaction centers and the associated electrochemical proton gradient across the thylakoid membrane result in a significant driving force for excess membrane dynamics. These observations provide a deeper understanding of the relationship between photosynthesis and cellular architecture.

Cyanobacteria dominated Earth’s biosphere for billions of years and are the reason for the oxygen atmosphere on our planet^1^. Thylakoid membranes in cyanobacteria are not found in chloroplasts as in plants and eukaryotic algae^2^, but typically occupy the peripheral region of the cell interior^3^. In *Synechocystis* sp. PCC 6803, the thylakoid membranes form concentric layers that tend to follow the shape of the cell envelope. Electron tomography^4^ and other high-resolution structural studies like super-resolution microscopy with fluorescent tagging^5^, fluorescence recovery after photobleaching^6^ and NMR spectroscopy^7^ have given reliable information about thylakoid membrane structure and architecture within cells.

Among non-invasive techniques, Small Angle Neutron Scattering (SANS) in combination with other methods^8^,^9^,^10^,^11^,^12^ is able to provide spatially averaged structural information about membranes systems under different experimental conditions, in algal cells^8^,^9^,^10^,^11^ and in isolated thylakoid membrane sistems^11^,^12^. The SANS data in Liberton *et al*. (2013) showed that within wild type cyanobacterial cells, the thylakoid membranes are arranged in various architectures, with center-to-center distances between 630 Å and 110 Å. This complex spacing corresponds to membrane pairs of different organizations: from pairs with sufficient space to accommodate light-harvesting complexes (phycobilisomes) within the interthylakoidal space, up to very closely appressed thylakoid membrane pairs that cannot accommodate light-harvesting antennas. These findings were acknowledged by^11^ in their review article, and Nagy *et al*. (2011) observed similar architecture in eukaryotic algae and plant thylakoids^9^. Because SANS is a sensitive technique capable of detecting repeat distances in large populations of cells, even membrane architectures that are not readily seen in small samples of cells examined by TEM are revealed by SANS.

However, to date little information exists about the internal dynamics of thylakoids in terms of flexibility and mobility of the membranes in their native environment, since biophysical studies are typically carried out under defined conditions in dilute aqueous environments that do not reflect the complex crowded environment of a living cell. In this work, we observe the mobility of thylakoid membranes in live cyanobacterial cells by neutron spectroscopy. Neutron Spin-Echo^13^(NSE) is a high-resolution spectroscopic technique that has already proved to be a successful method of studying the structure and dynamics of model bilayer lipid membranes^14^,^15^,^16^,^17^ and proteins in solution^18^ but has thus far not been used to study the internal dynamics of intact living cell components. This technique allows simultaneous observation of correlation times and length scales and thereby can directly deduce the geometry and modes of motion observed, such as membrane undulation or diffusion, which are not possible using purely energy-resolved spectroscopic techniques.

## Results and Discussion

### Assessment of the internal dynamics of thylakoids

We used the NSE spectrometer at the Spallation Neutron Source^19^ to study wild type cyanobacterial cells in a heavy water (deuterium oxide) solution. The high content of deuterium (D) in the heavy-water-filled spaces of the cyanobacterium provides excellent neutron contrast for the hydrogen-rich (H) lipid membranes and, therefore, the correlations between neighboring membranes dominate the coherent scattering signal in our probed window of observation, as shown previously^8^,^9^,^10^,^11^,^12^. Neutron data were collected at 20 °C during 12-hour light and dark alternating cycles to mimic the circadian clock. NSE measures the so-called intermediate scattering function which represents pair-correlation distances observed in reciprocal space (as in diffraction) and the time-dependent loss or relaxation of such correlations due to the mobility of the correlated spatial features. The reciprocal space coordinate or scattering vector magnitude, *q*, is inversely proportional to distances, *d*, in real space as *d* = 2π/*q*, and the corresponding time information is expressed as either relaxation times or inversely as relaxation rates *Γ*.

**Figure 1:**
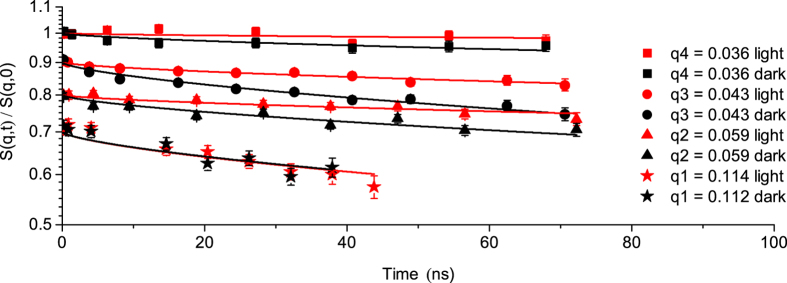
S(q, t)/S(q, 0) of cyanobacteria at 20 °C for light and dark states. All scattering functions start at unity and have been shifted for better visualization. Solid lines represent the stretched exponential function fitting with stretching exponent of 2/3^20^; 0.035 Å^−1^, 0.05 Å^−1^ and 0.095 Å^−1^ minimum value of momentum transfer were measured according to the correlation peaks observed in the SANS experiment^8^. Five different groupings of the time channels were applied, leading to 15 *q* values. Only four *q* values for each state are shown here in relation to Figs 2 and 3.

**Figure 2:**
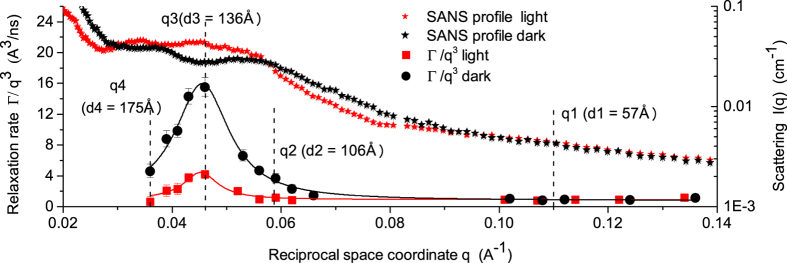
*q*^3^ dependence of the decay rate Γ for cyanobacteria during light and dark states. The vertical dashed lines show the position of the corresponding observed distances within membrane pairs, with *q*3 at the peak of Γ function. With increasing distance observed (from *q*1 to *q*2), the influence of the neighboring membrane becomes apparent in the increase of the relaxation rate up to a maximum where the membranes repel each other. This leads to a decrease of the relaxation rate to the state where the influence of the neighboring membrane is no longer visible (*q*4). Small Angle Neutron Scattering profiles^8^ are super-imposed to observe the correlation of dynamics features with the structure.

The normalized intermediate scattering functions of cyanobacteria (Fig. 1) show identical relaxation modes for both light and dark states at *q* values higher than 0.07 Å^−1^ (*d* ≤ 90 Å). Conversely, at *q* values lower than 0.07 Å^−1^ (*d* ≥ 90 Å) considerable difference exists between the two states, with slower decay in measurements collected in light compared to measurements collected in dark. NSE data were fitted by a stretched exponential function with a stretching exponent of 2/3, as predicted by Zilman & Granek^20^ for bilayer membranes (Table 1 and Methods). The spatial or *q* dependence of the relaxation rate Γ displayed in Fig. 2 is the major result of this study since its variations describe the dynamics of our system. Starting from the right side of Fig. 2 (high *q* values), a *q*^3^ dependence of the relaxation rate Γ is observed, which is a typical signature of the undulation motions in lipid bilayer membranes^21^ at length scales much smaller than the inter-membrane distance (the interthylakoidal space). In general, the tendency of decreasing magnitude of Γ with decreasing *q* is a common observation, as relaxations related to larger distances tend to take longer times. The absolute value of the relaxation rate at these high *q* values (short correlation distances) is similar for both light and dark measurements. More interestingly, we also observe a strong deviation from the *Γ(q*^3^) dependence when increasing the observed correlation distances to *q* values below 0.07 Å^−1^ (*d* ≥ 90 Å), peaks region in Fig. 2. This strong deviation from *q*^3^ dependence represents significant excess mobility that exists in addition to the underlying *q*^3^ dependent undulation dynamics. Between 0.07 Å^−1^ and 0.03 Å^−1^, Γ increases up to a maximum value at around 0.046 Å^−1^ and then decreases again slowly. This corresponds to a speed-up of the internal motion up to a very fast relaxation (peak in Γ relaxation rate represents a minimum of the relaxation time) followed by a slow-down process. This effect is more pronounced during the dark cycle compared to the light cycle.

The *q*^3^ dependent bending undulations describe correlations within a single membrane and are controlled by the elastic modulus with a higher bending coefficient or membrane stiffness corresponding to faster relaxation rates. From the *q*^3^ dependence of the relaxation rate Γ at high *q* values effective bending coefficients of 7655*k*_B_*T* for membrane structure in light and 7304*k*_B_*T* for membrane structure in dark were calculated^20^ (Table 1). The calculated bending coefficients show that during light measurement the membrane is stiffer compared to the dark state in which the bending coefficient shows a more flexible system. This is in agreement with a previous study on chloroplast remodeling within living cells of *Chlamydomonas reinhardtii*, which reports more stacked thylakoids with well-defined periodicity during the light harvesting state in comparison with a dark state in which the thylakoids are partially unstacked and undulated due to physiological responses of thylakoids to anaerobiosis^22^.

### Correlation of dynamics with membrane flexibility

**Figure 3:**
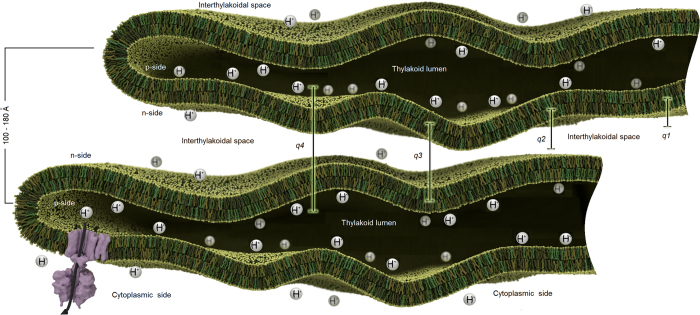
Thylakoid membrane sheet-like structure dynamics. A small region of two adjacent closely appressed membranes that cannot accommodate phycobilisomes is presented undulating freely in the cytoplasm, and the corresponding interthylakoidal distances. From *q*1 to *q*4 NSE samples a larger distance (57 Å–175 Å) within the interthylakoidal space and different relaxation behavior is observed. The excess of protons (H^ + ^) in the thylakoid lumen results in restricted membrane dynamics observed during light. At dark, H^ + ^ pressure is alleviated by chemiosmosis and the membranes undulate freely resulting in higher relaxation rates and excess dynamics. Purple feature is the ATP-Synthase responsible for moving down the electrochemical gradient. The center-to-center distance of a membrane pair corresponds to SANS correlation peaks for closely appressed membranes in Fig. 2 (100 Å–180 Å).

For model lipid bilayers, dynamic modes in addition to undulation have been interpreted as thickness fluctuations^14^,^15^,^16^,^17^. We propose a different explanation for our observations in cyanobacteria. The additional dynamics appear in the range of average length between 100 Å–180 Å that is rich with scattering intensity arising from correlations between very closely appressed thylakoid membrane pairs that cannot accommodate light harvesting antennas, as shown by^8^,^11^ and observed also by^10^ for other algal cells. The dynamics at this length scale therefore should contain strong contributions from the relative motion of neighboring membranes across the interthylakoid space (Fig. 3). In contrast to the *q*^3^ dependent bending undulations of individual membranes, motions of membrane pairs relative to each other in the direction across the interthylakoid space (large correlation distances) may be thought of as the local observation of a diffusive motion of large membrane areas. This diffusive component of the membranes motion is only observable through the time-dependent decay of correlations between membranes, and would not appear in the coherent signal of isolated single membranes. Importantly, although a stiffer membrane, submitted to a random undulation, relaxes faster to its flat state, this membrane is less efficient in exploring the available volume, in our case the interthylakoid space. The same space is sampled faster by a flexible membrane structure that is very efficient in exploring the available volume^20^ (Table 1). The membrane exhibits a speed-up/slow-down of undulation motion with different amplitude when two adjacent membranes feel each other’s influence in light or dark. Softer thylakoid membranes in the dark display three-to four-fold excess dynamics compared to membranes under light conditions. The more rigid membrane in light takes a longer time to relax which is the time necessary for the membrane structure to move through the available space. This represents the relaxation times we observe by NSE of about 2000 ns–3000 ns in light and only 600 ns–800 ns in the dark, for the peak position in Fig. 2.

### The relation between excess dynamics and physiological processes within thylakoids

There could be several possible explanations for the difference in membrane mobility observed between light and dark. One reason could lie in the function of the photosynthetic apparatus in the thylakoid membrane. Absorption of light by photosystem II initiates water oxidation that results in transfer of photo-generated reducing equivalents to photosystem I. This results in an excess of protons in the lumenal space of the thylakoid membrane (Fig. 3) that is alleviated by moving an electrochemical gradient (H^+^) through the membrane by chemiosmosis^23^. In light, photosynthesis maintains the presence of a proton concentration gradient. The increased density of protons within the lumenal space could result in increased pressure upon membranes. Such pressurized membranes will appear stiffer when observed during light and display restricted motility. The protons move across the thylakoid membrane by the function of ATP-Synthase. They are the motive force to generate ATP. In the dark cycle when light harvesting and photosynthesis stop, the difference in the proton density between the lumenal and the interthylakoidal space is finally, equilibrated over time. Pressure is released, and the membrane becomes more relaxed and flexible and can move easily within the available space.

Furthermore, refinements to the chemiosmotic hypothesis have proposed that localized domains of protons are confined to the region near the thylakoid membrane, rather than being delocalized within the lumenal space^24^. Such organization suggests that in addition to the increased pressure by the presence of protons, the inner leaflet of the thylakoid membrane bilayer (the p-side, Fig. 3) has a higher positive charge than the outer leaflet (the n-side). The negative outer leaflets would therefore repel each other when the undulating motion brought two neighboring membranes in close proximity. This repelling will constrict the excess undulation motion during light. The electrochemical gradient is dissipated by chemiosmosis. The repelling will stop and the membranes recover their higher flexibility during dark when the proton density equilibrates.

Another explanation of the observed differences in membrane excess dynamics between light and dark could be the dynamic control of protein diffusion within the thylakoid lumen^25^. In higher plant thylakoids, the lumenal space undergoes a significant expansion in light. In a study involving electron microscopy, light scattering and theoretical modeling of the lumenal space in *Arabidopsis*, it was shown that reversible changes in the thylakoid lumen induced by alteration of the light environment would modulate the diffusion of proteins within the restricted space of the lumenal compartment. This is done in order to facilitate PC-mediated electron transport and to repair photo-damaged photosystem II complexes^25^. If similar effects are present in cyanobacterial thylakoids, then the expansion of the lumen restricts the undulation motions of the membranes, i.e. membranes observed during light will appear again stiffer.

Is not certain whether the observed changes may constitute a mechanism by which cyanobacteria regulate photosynthetic activity in response to light intensity, or may be a byproduct of photosynthetic activity, e.g., through charge gradients across lipid membranes, or through an unknown, more indirect chain of events. However, this study is the first direct measurement of the undulation dynamics of photosynthetic membranes in living prokaryotic cells and adds to the open question of whether other components of the photosynthetic machinery exhibit internal dynamics and how these dynamics might affect the yield of photosynthesis. The question of how the plasticity and dynamics of thylakoid membranes can be regulated, and related to the functions of sub-cellular architectures, is now an open field in biological research. This holds promise for important discoveries and we are giving a first demonstration of the possibility to observe directly such complex dynamic relations.

## Methods

### Sample preparation

*Synechocystis* sp. PCC 6803 was grown in BG11 medium at 20 °C and 30 μmole photons·m^-2^·s^−1^ light intensity. The cells were exchanged into a D_2_O based BG11 medium by three sequential centrifugation and resuspension steps^8^. For the final resuspension step the cells were suspended in 4 ml of D_2_O-based BG11 media (OD_750nm_ = 41.6, 1 cm pathlength). A control sample from the same culture was maintained under conditions identical to the NSE sample (OD_750nm_ = 36.5, 1 cm pathlength). There was no significant change in the OD_750nm_ of the sample over the time course of the experiment.

### Neutron Spin Echo experiment

NSE experiments were performed using the NSE spectrometer at the Spallation Neutron Source^19^, Oak Ridge National Laboratory. Measurements were carried out in 4 mm-path quartz cells. A closed sample environment was used to allow dark adaptation of the samples. The sample holder was equipped with a lighting apparatus that illuminated the samples with cool white LED lights set at 100 μmole photons·m^−2^·s^−1^. The NSE experiment was performed at incident wavelengths of 9 Å and 11 Å accessing a dynamical range of 0.1 ≤ *τ*_max_ ≤ 130 ns at different momentum transfers for each sample according to correlation peaks observed in the SANS experiment^8^. Solid angles of 0.035 Å^−1^, 0.05 Å^−1^ and 0.095 Å^−1^ were measured (minimum value of momentum transfer). NSE spectra were taken at 20 °C under 12 h light and dark alternating cycles to mimic the circadian clock. One hour of equilibration was allowed between each cycle so that cells may adapt to the light or dark condition before data collection. Graphite foil was used as a standard elastic reference and solvent BG11 measurements were also necessary for proper data reduction. The entire experiment lasted 14 days: 7 days for cyanobacterial sample and 7 days for reference and background sample.

### Data analysis

The data analysis was performed with the standard reduction software package of the NSE-SNS instrument called ECHODET. Data were acquired with sufficient statistics so the entire wavelength spectra could be divided in 5 different solid angles for a better discretization of the *q* dependence. The intermediate scattering functions were fitted by a stretched exponential function with a stretching exponent of 2/3 predicted by Zilman & Granek^20^ for single membrane (Fig. 1):


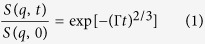


were Γ is the relaxation rate. Using this approach for fitting data one might argue that the translational diffusion of the entire cyanobacteria cell has also a contribution to the overall dynamics of the system. However, the cyanobacteria cell has a diameter of about 3.8 – 5.2 μm and the diffusion coefficient of such a large cell is outside the NSE window we can access for measurements. Therefore, translational diffusion was neglected from further analysis. To account for the bending motion of the membranes we applied the single membrane fluctuation model of Zilman & Granek^20^. This model, based on mode coupling theory, explains the dependence with *q*^3^ of the relaxation rate for systems where the hydrodynamic interactions dominate and the wavelengths are shorter than the characteristic correlation length, in our case *1/q* « interthylakoidal distance^21^:


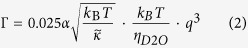


with 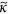 as the effective bending modulus; *α* is a scaling factor equal unity; *k*_B_*T* is the thermal energy; *η*_D2O_ is the viscosity of the solvent at *T* = 20 °C. Γ/*q*^3^ represents in fact the linear dependence observed at *q* values higher than 0.07 Å^−1^ (*d* ≤ 90 Å) in Fig. 2. The calculated values of bending coefficients are displayed in Table 1.

## Additional Information

**How to cite this article**: Stingaciu, L.-R. *et al*. Revealing the Dynamics of Thylakoid Membranes in Living Cyanobacterial Cells. *Sci. Rep*. **6**, 19627; doi: 10.1038/srep19627 (2016).

## Figures and Tables

**Table 1:** Characteristics of membrane relaxation and bending fluctuations.

Sample	q (Å^−1^)	Γ/q^3^ (Å^3^/ns)	Γ (1/ns)	τ (ns)	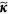 (k_B_T) ^**^
PCC6803 light	0.114	0.92	1.37E-3	729.9	7655.45
PCC6803 dark	0.112	0.94	1.33E-3	751.9	7304.45
PCC6803 light	0.059	1.19	2.44E-4	4098.3	–
PCC6803 dark	0.059	3.69	7.59E-4	1317.5	–
PCC6803 light	0.043	3.75	2.98E-4	3355.7	–
PCC6803 dark	0.043	14.34	1.14E-3	877.2	–
PCC6803 light	0.036	0.61	3.35E-5	29850.7	–
PCC6803 dark	0.036	4.61	2.29E-4	4366.8	–

A value of 0.00125Kg·m·s^−1^ was use as D_2_O viscosity at 20 °C for calculation of bending modulus. ^**^Effective bending coefficient according to Zilman & Granek^20^ are valid when calculated only in the high *q* regime where 1/*q* « interthylakoidal distance (the linear dependence around *q*1 position in Fig. 2).
